# 1359. Reported Neurologic, Ocular, and Otic Manifestations among Syphilis Cases — 16 States, 2019

**DOI:** 10.1093/ofid/ofab466.1551

**Published:** 2021-12-04

**Authors:** David A Jackson, Robert McDonald, Hillard Weinstock, Elizabeth Torrone

**Affiliations:** 1 Centers for Disease Control and Prevention, Atlanta, Georgia; 2 Centers for Disease Control and Prevention, New York State Department of Health, Atlanta, GA

## Abstract

**Background:**

Syphilis can cause neurologic, ocular, or otic manifestations at any stage, possibly resulting in permanent disability or even death. In 2018, CDC began collecting clinical manifestation data for syphilis cases reported through the National Notifiable Diseases Surveillance System (NNDSS). We present the first estimates of the prevalence of neurologic, ocular, and otic manifestations among syphilis cases in the United States.

**Methods:**

We reviewed NNDSS data to identify jurisdictions (states + DC) who reported ≥ 70% of their syphilis cases with clinical manifestation data (considered to have “complete reporting”) in 2019. Among these jurisdictions, we determined the prevalence of neurologic, ocular, and otic manifestations (combining verified, likely, and possible clinical manifestations together), stratified by HIV status and by syphilis stage (Unknown/late syphilis vs. Early syphilis [Primary, Secondary, and Early non primary non secondary syphilis]).

**Results:**

In 2019, 16 states had complete reporting for neurologic, otic, and ocular manifestations. Of the 41,216 syphilis cases reported in these jurisdictions, clinical manifestations were infrequently reported: neurologic (n=445, 1.1%), ocular (n=461, 1.1%), and otic (n=166, 0.4%). Prevalence was higher among HIV-infected persons compared to HIV-negative persons for neurologic (1.4% vs. 0.9%) and ocular manifestations (1.3% vs 1.0%) but was similar for otic manifestations (0.4% vs 0.4%). Prevalence was higher among persons diagnosed with Unknown/late syphilis compared to Early syphilis for neurologic (1.6% vs 0.8%) and ocular manifestations (1.6% vs 0.9%) but similar for otic manifestations (0.5% vs 0.4%); however, 49.4% of cases reported with ≥ 1 of these clinical manifestations were diagnosed with Early syphilis.

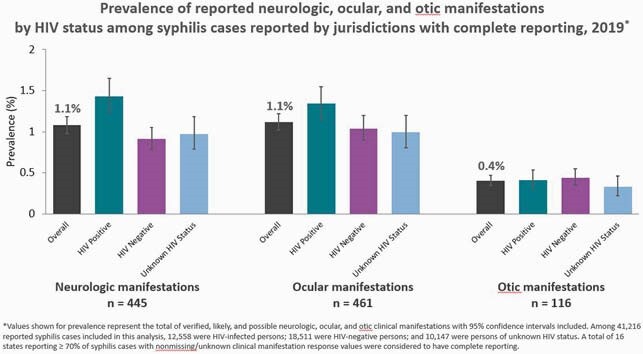

**Conclusion:**

The prevalence of neurologic, ocular, and otic manifestations was low among syphilis cases, but case data likely underestimate the true burden given potential underreporting. The frequency of clinical manifestations, including among HIV-negative persons and persons diagnosed with Early syphilis, emphasizes the importance of evaluating all syphilis cases for clinical signs or symptoms regardless of stage or HIV status.

**Disclosures:**

**All Authors**: No reported disclosures

